# Targeting GPCRs to treat cardiac fibrosis

**DOI:** 10.3389/fcvm.2022.1011176

**Published:** 2022-10-06

**Authors:** Hao Zhang, Lu Ren, Rabindra Vishwadev Shivnaraine

**Affiliations:** ^1^Department of Medicine, Division of Cardiovascular Medicine, Stanford Cardiovascular Institute, Stanford University, Stanford, CA, United States; ^2^Department of Molecular and Cellular Physiology, Stanford University, Stanford, CA, United States

**Keywords:** cardiac fibrosis, GPCR, antifibrotic therapy, fibroblast, G protein, crosstalk

## Abstract

Cardiac fibrosis occurs ubiquitously in ischemic heart failure, genetic cardiomyopathies, diabetes mellitus, and aging. It triggers myocardial stiffness, which impairs cardiac function, ultimately progressing to end-stage heart failure and increased mortality. Although several targets for anti-fibrotic therapies have been identified, including TGF-β and receptor tyrosine kinase, there is currently no FDA-approved drug specifically targeting cardiac fibrosis. G protein-coupled receptors (GPCRs) are integral, multipass membrane-bound receptors that exhibit diverse and cell-specific expression, offering novel and unrealized therapeutic targets for cardiac fibrosis. This review highlights the emerging roles of several GPCRs and briefly explores their downstream pathways that are crucial in cardiac fibrosis. We will not only provide an overview of the GPCRs expressed on cardiac fibroblasts that are directly involved in myofibroblast activation but also describe those GPCRs which contribute to cardiac fibrosis via indirect crosstalk mechanisms. We also discuss the challenges of identifying novel effective therapies for cardiac fibrosis and offer strategies to circumvent these challenges.

## Pathophysiology of cardiac fibrosis

Cardiovascular diseases (CVD), which affect the heart or vasculature, remain the leading cause of mortality, responsible for ~31% of all deaths worldwide (1). Fibrotic diseases lead to nearly 1 million deaths annually, most of which are due to lung and cardiac fibrosis (2). Different types and consequences of cardiac fibrosis exist, and their pathology depends on the underlying cause. For example, aging, hypertension, and diabetes induce myocardial interstitial fibrosis and decrease ventricular compliance, leading to the pathogenesis of heart failure with diastolic dysfunction (3, 4). However, fibrosis is not invariably detrimental. In myocardial infarction (MI), the loss of a substantial number of cardiomyocytes triggers myofibroblasts activation, representing reparative fibrosis and contributing to scar formation (5). Although the scar lacks contractile ability, it serves an important protective role in keeping cardiac structural integrity and preventing catastrophic mechanical complications, such as cardiac rupture (6).

Cardiac fibroblast is an abundant cell type in the heart (7). In fibrosis diseases, cardiac fibroblasts are activated to myofibroblasts (MyoFB), which become highly proliferative and specialized in the generation of extracellular matrix proteins and contractile genes, such as ACTA2, encoding for smooth muscle α-actin (8, 9). Histologically, cardiac fibrosis is identified as the enlargement of the cardiac interstitium resulting from the deposition of extracellular proteins such as collagen, fibronectin, and elastin (3). This accumulation accompanies most cardiac pathologic conditions, such as myocardial stiffness and diastolic dysfunction, and the extent of cardiac fibrosis is a predictor of adverse outcomes (3).

Although there is no FDA-approved drug specifically targeting cardiac myofibroblast activation, pirfenidone and nintedanib have been approved to treat idiopathic lung fibrosis (10). In addition, pirfenidone has been tested in Phase II clinical trial “PIROUETTE” among patients with heart failure with preserved ejection fraction (HFpEF) and myocardial fibrosis (11). The results show that the administration of pirfenidone for 52 weeks reduced myocardial fibrosis (12), indicating fibrosis shares similar pathways, and antifibrotic therapy can be utilized universally among different organs.

## GPCRs signaling in heart tissue

G protein-coupled receptors (GPCRs), located at the cell membrane, possess diverse expression profiles throughout the body and are known to exhibit tissue and cell-specific membrane expressions (13). The basic function of GPCRs is to transduce extracellular stimulus into intracellular signals (14). The “G-proteins” that coupled to GPCRs are composed of a heterotrimer of α, β, and γ subunits—Gα, Gβ, and Gγ, respectively—that is kept in an inactive basal state where α*βγ* are tightly bound and activated when α dissociates from βγ (14). Since GPCRs consist of more than 800 receptors and account for the most diverse family of proteins in the human genome, the combination of GPCRs at a particular cell type may be substantially heterogeneous and diverse (15). In this review, we will focus on the roles of GPCR and the downstream signaling in cardiac fibrosis regulation and illustrate their utility as promising targets for cardiac fibrosis (Table 1).

**Table 1:** Representative GPCRs and downstream pathways involved in fibrosis regulation.

**Receptor family**	**Target**	**Transduction mechanism**	**Drug**	**Preclinical and clinical studies**
**Part 1: GPCRs expressed on fibroblasts**				
Protease-activated receptor	PAR1	Gαi/o, Gαq/11, and Gα12/13	PAR1 antagonist: SCH79797	Inhibit cardiac fibrosis in renin-overexpressing hypertensive mice model (16).
Protease-activated receptor	PAR2	Gαi/o, Gαq/11, and Gα12/13	PAR2 antagonist: PZ-235	Inhibit liver fibrosis in non-alcoholic fatty liver disease mice model (17); In contrast, PAR2 knockout increases cardiac fibrosis due to compensatory augmented PAR1 (18).
Lysophospholipid receptor	LPA1	Gαi/o, Gαq/11, and Gα12/13	LPA1 antagonist: BMS-986020	Inhibit cardiac fibrosis in a hypertrophic cardiomyopathy mouse model (19); Effective in Phase II clinical trial for idiopathic pulmonary fibrosis (NCT01766817) (20).
Lysophospholipid receptor	S1PR1	Gαq/11, and Gα12/13	/	Overexpression induces cardiac fibrosis (21).
Adenosine receptor	A2B	Gαs and Gαq	A2B antagonist: GS6201	Inhibit cardiac fibrosis in myocardial infarction mice model (22).
Adenosine receptor	A1	Gαi	A1 antagonist: SLV320	Reduce myocardial fibrosis in rats with nephrectomy (23).
Adenosine receptor	A2A	Gαs	A2A agonist: CGS21680	Reduce cardiac fibrosis in DOCA-salt treated mice (24).
Prostaglandin receptor	EP4	Gαs	EP4 agonist: ONO-0260164	Inhibit cardiac fibrosis in pressure overload-induced mice model (25).
**Part 2: GPCRs expressed on cardiomyocytes and endothelial cells**				
β adrenergic receptor	β1AR	Gαs	β blocker	Inhibit maladaptive remodeling and cardiac fibrosis *via* cardiomyocyte-fibroblast crosstalk (26).
Angiotensin receptor	AT1R	Gαq/11	AT1R inhibitors and antagonist: ACEI/ARB	Inhibit adverse cardiac remodeling and fibrosis in heart failure patients *via* direct effects on fibroblasts and indirect effects *via* crosstalk (27).
Endothelial receptor	ET_A_	Gαq	/	ET-1 knockout exerted antifibrotic effects in the diabetes mice model by inhibiting endothelial-to-mesenchymal transition (28).
**Part 3: Targeted G protein and downstream pathways**				
-	-	Gβγ	Gβγ inhibitor: Gallein	Inhibit cardiac fibrosis in myocardial infarction mice model (29).
-	-	cAMP	adenylyl cyclase activator: forskolin	Elevate cAMP inhibits myofibroblast activation in cardiac fibroblasts (30).
-	-	YAP/TAZ	YAP inhibitor: verteporfin	Inhibit cardiac fibrosis in myocardial infarction mice model (31).

GPCR signaling may also significantly vary within the cell. To date, 21 Gα, 6 Gβ, and 12 Gγ have been discovered (14). Heterotrimeric G proteins are generally identified by their Gα subunits and classified into four major groups: Gα stimulatory (Gαs), Gα inhibitory (Gαi), Gαq, and Gα12/13 (14). Different G-protein subfamilies execute distinct signaling cascades. Gαs stimulates adenylyl cyclase to increase the second messenger cyclic adenosine monophosphate (cAMP), which results in protein kinase A (PKA) activation and subsequent phosphorylation of intracellular proteins (32). Conversely, Gαi exhibits a suppressive effect on adenylyl cyclase, decreasing intracellular cAMP (32). Gαq activates phospholipase C (PLC), resulting in phosphatidylinositol 4,5–bisphosphate (PIP_2_) cleavage and producing the second messengers inositol 1,4,5–triphosphate (IP_3_) and diacylglycerol (DAG). IP_3_ triggers Ca^2+^ release from the endoplasmic reticulum, along with DAG to activate protein kinase C (PKC) (33). Gα12/13 can activate the small GTPase Rho, which serves as a regulator of a number of intracellular processes, including actin stress fibers formation and cell growth controller (34). GPCRs exist widely and heterogeneously in a cell-specific distribution among the three major cell types found within the heart (i.e., cardiomyocytes, endothelial cells, cardiac fibroblasts) (35, 36).

Besides the G protein signaling cascades, β-arrestins have the capacity to regulate GPCR activity independently (37). Previously, β-arrestins were discovered to mediate receptor internalization and desensitization, exemplified by the role of β-arrestin in β1 adrenergic receptors (β1AR) desensitization in cardiomyocytes (38). Later, other functions of β-arrestins were identified as signal transducers to regulate a variety of intracellular signaling pathways (39), including transforming growth factor-β (TGF-β), as well as downstream kinases such as mitogen-activated protein kinase (MAPK) and phosphoinositide 3-kinase (PI3K) (40), which are highly involved in the regulation of fibrosis.

The GPCR expression level depends on a steady-state transport of receptors between the cell membrane and endosomes (41). Agonists of GPCRs induce internalization that alters the membrane expression level in the short term. Prolonged over-stimulation leads to a downregulation of these receptors at the transcriptional level (41). For example, β1AR and β2AR are the predominant GPCR subtypes expressed in cardiomyocytes and the primary regulators of cardiovascular function (42). Epinephrine stimulates β2AR and initially activates Gs, which increases the beating rate. However, after 10–15 min of stimulation, β2AR signals predominantly through Gi, which decreases the contraction rate (43). Therefore, pathophysiological states may alter not only the expression of these receptors, but also the coupling specificity between the receptors and G proteins (41).

The diverse and cell-specific membrane expression of GPCRs, combined with their important roles in pathophysiology, make them frequent therapeutic targets, leading to ~35% of all FDA-approved drugs (13). A variety of GPCRs have been implicated in the pathogenesis of fibrosis (44, 45). Although several earlier drug discovery campaigns have targeted GPCRs to treat fibrosis, effective treatment is still lacking (44). As fibroblasts among different organs share similar features (46), we will discuss the common and distinct features of those GPCRs involved in cardiac fibrosis compared to fibrosis in other organs.

## GPCRs expressed on cardiac fibroblasts

### PAR receptors

**Figure 1:**
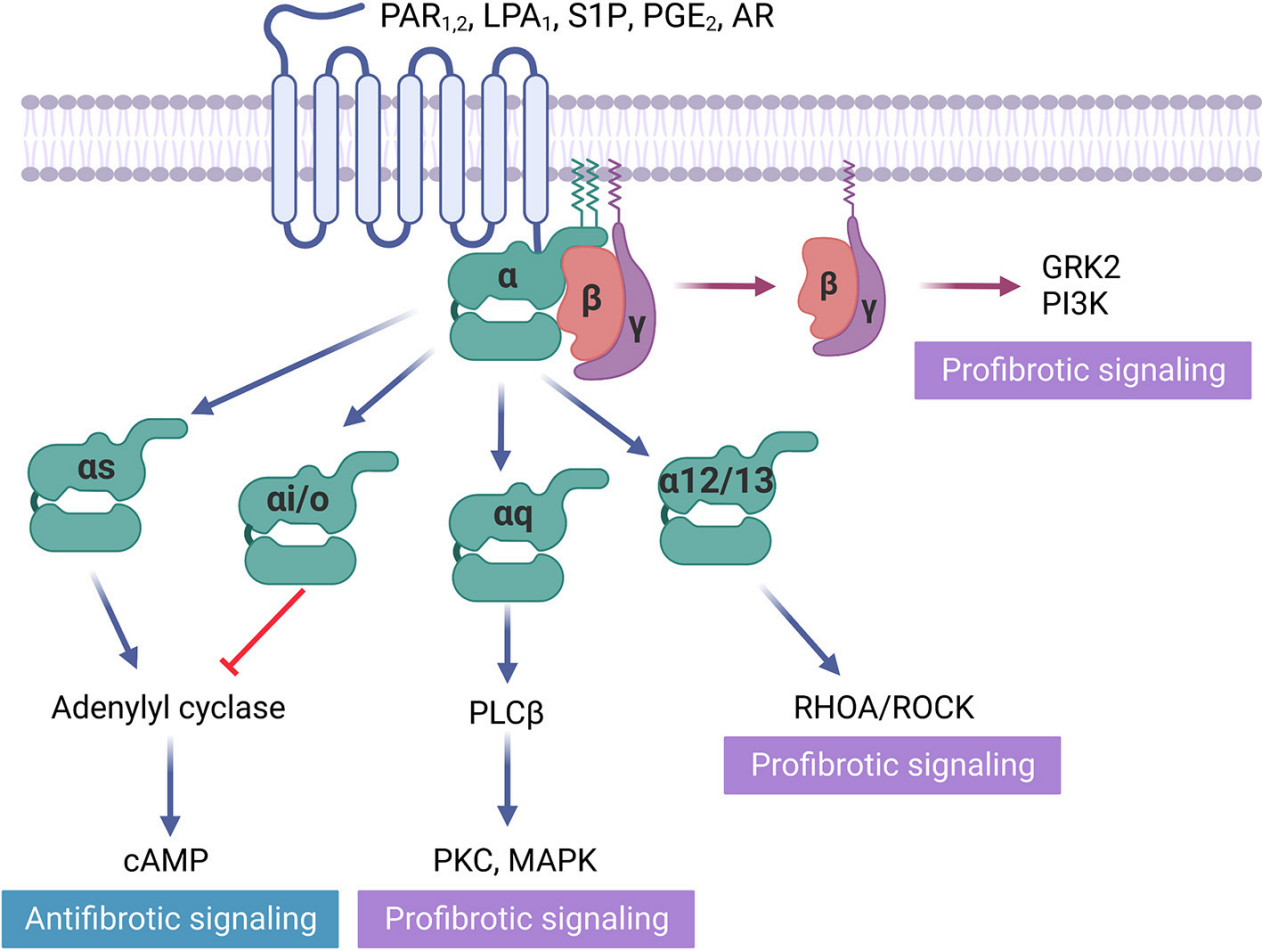
GPCRs in fibrosis regulation. Some GPCRs mediate profibrotic signals, including protease-activated receptors (PAR1), lysophospholipid receptors (LPA1, S1P), and adenosine receptors (AR), that activate receptors coupled to Gαi/o, Gαq/11, Gα12/13. Gβγ activation is also proved to be profibrotic. In contrast, prostaglandin receptor (PGE2), which activates receptors Gαs and direct cAMP activator exert antifibrotic effects.

Protease-activated receptors (PAR) become activated from specific cleavage of the amino-terminal sequence that consequently exposes a new N-terminal sequence functioned as a tethered ligand (47) (Figure 1). Activation of protease-activated receptor-1 (PAR1), which couples to Gq/11, Gi/o, and G12/13, elevates the level of profibrotic gene expression in rat cardiac fibroblasts, leading to myofibroblast activation and increasing collagen synthesis by 60% (48). Conversely, inhibiting PAR1 with SCH79797 attenuates cardiac fibrosis and hypertrophy in the renin-overexpressing induced hypertensive mice model (16). However, PAR1 is highly expressed in platelets, and its antagonist is used for antiplatelet therapy (49). The off-target effect restricts the application of this target for fibrosis treatment. Protease-activated receptor-2 (PAR2), which also couples to Gq/11, Gi/o, and G12/13, is expressed on liver stellate cells that regulate the response to liver cirrhosis (17). PAR2 inhibitor PZ-235 has significantly suppressed liver fibrosis and collagen deposition up to 50–100% (17). In contrast, 1-year-old PAR2-knockout mice suffered from diastolic dysfunction, associated with an increased α-SMA, collagen deposition, lysyl oxidase activity, and collagen cross-linking. The absence of PAR2 contributed to an augmented profibrotic PAR1 and dependent signaling in heart (18), indicating that the same GPCR may exert a distinct role in fibrosis regulation among different organs.

### Lysophospholipid receptors

The lysophospholipid receptor group is a member of the GPCR family of integral membrane proteins essential for lipid signaling (50). Lysophosphatidic acid (LPA) and its G protein-coupled receptor (LPA1) contribute to fibrosis progression (51). LPA1 is highly expressed on human primary fibroblasts, which predominantly couples to Gαq/11, Gαi/o, and Gα12/13 proteins (52). Myocardial fibrosis is a key pathologic feature of hypertrophic cardiomyopathy (HCM). In the HCM mouse model, LPA1 ablation developed significantly less hypertrophy and fibrosis (19). Since LPA1 antagonist BMS-986020 has been tested in Phase II clinical trial for idiopathic pulmonary fibrosis (20), it could potentially be used to treat cardiac fibrosis in the future. Sphingosine 1 phosphate receptor-1 (S1PR1), which also belongs to the lysophospholipid receptor family, couples to Gαq/11, Gαi/o, and Gα12/13. Its overexpression was capable to induces cardiac hypertrophy and fibrosis through angiotensin II and interleukin-6 in S1PR1-transgenic mouse heart (21).

### Adenosine receptors

In addition to adenosine and its receptor's crucial role in wound healing (53), they have also been found to promote fibrosis by producing excess matrix in the heart, skin, lungs, and liver (54). The adenosine receptor (AR) family comprises four GPCRs: A1, A2A, A2B, and A3 (55). Previous studies have suggested AR antagonists as an effective fibrosis treatment. For example, caffeine, a non-selective adenosine receptor antagonist, has been shown to alleviate liver fibrosis in animal models and to reduce liver fibrosis in patients with chronic hepatitis C (56, 57). However, the weak affinity of caffeine to ARs requires micromolar concentration in plasma, which prevents its potential for drug development.

A pro-fibrotic role for the A2B, which couples to Gαs and Gαq, has been supported by a study using A2B knockout mice, which exhibited improved diastolic dysfunction and attenuated interstitial fibrosis 8 weeks after MI (22). Selective A2B antagonist, GS-6201, significantly reduced cardiac enlargement and dysfunction compared to vehicle in the mouse MI model (58). Similarly, in the rat model of myocardial ischemia-reperfusion, GS-6201 improved ejection fraction and decreased fibrosis in the non-infarct and border zones (59). In addition, the adenosine A1 receptor (i.e., a Gαi coupled receptor) antagonist SLV320 reduces myocardial fibrosis in rats with nephrectomy (23). In contrast, the activation of A2A (i.e., a Gαs coupled receptor) by CGS21680 reduced cardiomyocyte hypertrophy, cardiac inflammation, and fibrosis in deoxycorticosterone acetate (DOCA) treated mice (24). These interesting findings suggest the effects of ARs on myocardial adaptation are subtype-specific. The distinct role of AR subtypes in cardiac fibrosis regulation needs further investigation.

### Prostaglandin receptor

Prostaglandin E2 (PGE2) receptor, which couples to Gαs, acts *via* cAMP and is abundantly expressed in fibroblasts (60). Prostaglandin E2 receptor 4 (EP4) stimulation was reported to be cardioprotective. Its agonist ONO-0260164 significantly prevented systolic dysfunction and the progression of myocardial fibrosis in the mouse model of cardiac hypertrophy induced by transverse aortic constriction (25). Conversely, EP4 knockout mice exhibited concentric hypertrophy and myocardial fibrosis in mice fed with high-fat diet (61).

## GPCRs involved in cardiac fibrosis *via* crosstalk

**Figure 2:**
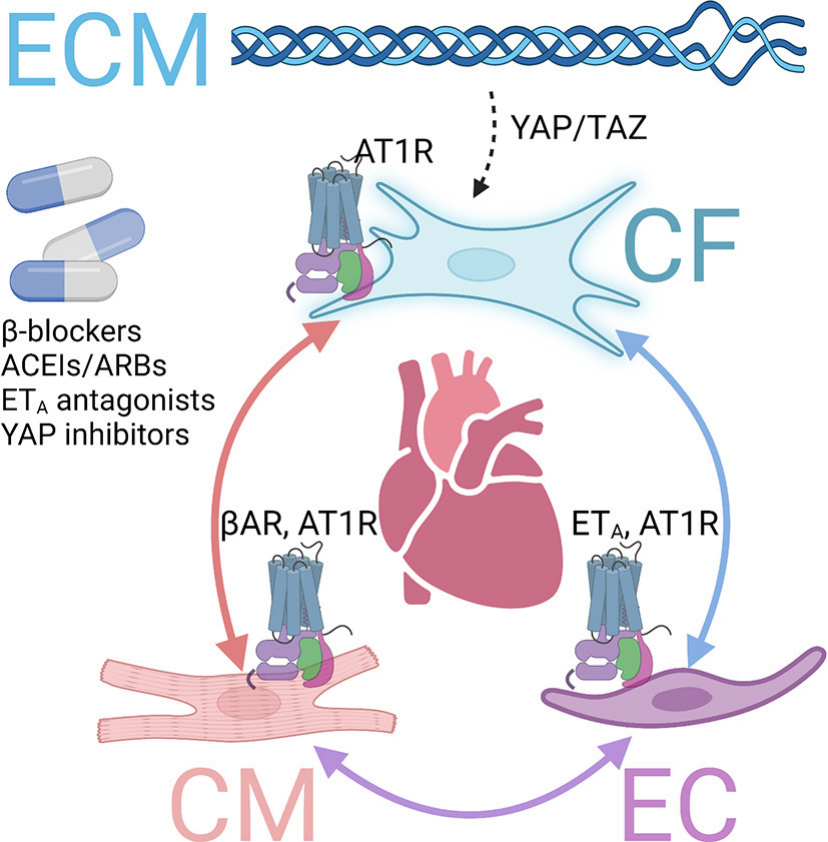
GPCRs involved in fibrosis *via* crosstalk mechanisms. Several GPCRs contribute to cardiac fibrosis *via* interactions among cardiomyocytes (CMs), endothelial cells (ECs), cardiac fibroblasts (CFs), and extracellular matrix (ECM). Inhibiting those and inhibiting those GPCRs or downstream pathways exert antifibrotic effects in the heart. βAR, β adrenergic receptor. AT1R, angiotensin receptor type 1. ET_A_, endothelin receptor A.

Of particular relevance to cardiac fibrosis compared to fibrosis in other organs are the distinct features of the heart tissue environment (4). In the heart, the crosstalk between fibroblasts, cardiomyocytes, and endothelial cells plays an important role in fibrosis regulation (62). Different types of intercellular crosstalk exist, including direct contact communication *via* gap junction or nanotubes, indirect cell interaction *via* paracrine factors, and cell-extracellular matrix (ECM) interaction (63). In particular, several GPCRs and downstream pathways have been involved in cardiac fibrosis regulation *via* the crosstalk mechanism (Figure 2).

### β adrenergic receptor

The stimulation of β1AR on cardiomyocytes increases contractility through PKA-mediated phosphorylation of specific regulatory proteins to increase intracellular Ca^2+^ level or sensitivity, such as L-type Ca^2+^ channels, phospholamban, ryanodine receptor, and troponin I (64). However, long-term stimulation of β1AR leads to cardiac hypertrophy and fibrosis, progressively developing into heart failure (26). Although β-blockers cannot directly prevent MyoFB activation, those drugs have been demonstrated to prevent cardiac fibrosis and improve survival in mice models and clinics *via* cardiomyocyte-fibroblast communications (26).

### Angiotensin II receptor

Angiotensin receptors, particularly AT1R, play an important role in heart pathophysiology (65). Cardiac AT1R, coupled to Gαq, is upregulated with hypertrophic and ischemia, promoting adverse maladaptive cardiac remodeling, including cardiac fibrosis in chronic heart failure (66). Ang II, the endogenous ligand, is a peptide hormone that regulates several critical physiological processes, representing a principal component of the renin-angiotensin-aldosterone system (66). AT1R is expressed in cardiomyocytes, endothelial cells, and cardiac fibroblasts. Its overexpression lead to cardiac fibrosis and hypertrophy, whereas knockout of AT1R improved cardiac function (67). The mechanisms of profibrotic AT1R are multifold, including direct effects on fibroblasts and, more importantly, *via* crosstalk between fibroblasts, cardiomyocytes, or endothelial cells (62). Due to its central role in cardiovascular pathophysiology, AT1R inhibitors and antagonists, ACEI and ARB, are essential drugs for heart failure treatment and have been proven to protect against maladaptive remodeling and cardiac fibrosis (27).

### Endothelial receptor

Among the endothelial receptors, ET_A_ is the major isoform found in the cardiovascular system (68). Chronic stimulation with ET_A_ agonist ET-1 is associated with adverse effects, including cardiac hypertrophy and fibrosis (4). High levels of ET-1 in plasma have been found in heart failure patients (68). ET_A_, which couples to Gαq, promotes IP formation and activates MAPK signaling (69). ET1 knockout exerted antifibrotic effects in the diabetes mice model *via* endothelial-fibroblast interaction (70).

### Fibroblast-ECM interaction

Increased ECM stiffness and tension are known to activate fibroblasts through the yes-associated protein 1 (YAP) and transcriptional coactivator with PDZ-binding motif (TAZ) signaling pathway, which mediates the mechanical sensing of cardiac fibroblasts in fibrotic diseases (71). The Hippo-YAP-TAZ pathway is mediated by multiple G proteins, and therefore involved in common GPCR downstream pathways (72). Fibroblast conditional deletion of Yap1 attenuated injury-induced cardiac fibrosis either after acute MI or chronic angiotensin II–phenylephrine stimulation (73).

## G protein and downstream pathways

Multiple profibrotic GPCRs are co-expressed in fibroblasts, indicating the possibility of redundancy of G protein activity in myofibroblast activation mechanisms. These results challenge the effectiveness of antifibrotic therapy by antagonizing one single GPCR. To address the redundancy issue, one strategy is to target the G protein itself or convergent downstream pathways.

### Gαs and cAMP

GPCRs coupled to Gαs activate adenylyl cyclase to elevate the intracellular cAMP level (14). To examine the consequence of Gαs signaling, forskolin is used as an activator of adenyl cyclase (AC) to increase cAMP. Experiments using forskolin to activate AC have identified that this pathway results in the inhibition of TGF-β-induced fibroblast activation (74). In rat cardiac fibroblasts, elevating cAMP formation by AC overexpression or by forskolin attenuates myofibroblast marker gene expression and collagen synthesis (30).

### Gβγ

G protein βγ subunits were identified as G protein components almost 30 years ago (75). Since then, Gβγ signaling has been demonstrated as diverse, regulating a number of downstream pathways depending on the interacting effectors. For example, Gβγ has the capacity to activate G protein-coupled inward rectifier K^+^ (GIRK) channels, phospholipase A and C, plasma membrane Ca^2+^ pump, PI3K-AKT, GPCR kinase 2 (GRK2), and guanine exchange factors (GEFs) for small G proteins (76). Moreover, a small molecule Gβγ inhibitor Gallein has been shown to inhibit myofibroblast activation *in vitro* (77). Gallein treatment a week after ischemic/reperfusion is effective in reducing cardiac fibrosis and preserving ejection fraction in a mouse model of chronic heart failure (29). Since GPCRs are comprised of α and βγ subunits, differentiating the effects of Gas (i.e., antifibrotic) from that of Gβγ (i.e., fibrotic) is challenging and may depend on factors such as cell type of receptor expression.

### Hippo-YAP pathway

As previously described, Hippo-YAP is the common downstream pathway of multiple G proteins (72). Specially, activation of lysophospholipid receptors, such as LPA and S1P, coupled simultaneously with Gαi, Gas, and Gα12/13, inhibit the Hippo signaling kinases 1/2 (Lats1/2) (72). The inhibition of Lats1/2 then stimulates the transcription coactivators YAP and TAZ, which are related to cell proliferation and migration (72). YAP and TAZ have recently been identified as promotors for myofibroblast activation in cardiac fibrosis (31). Interestingly, a previous study indicated that the suppression of YAP/TAZ signaling by lovastatin attenuates angiotensin II-induced cardiac fibrosis, both *in vitro* and *in vivo* (78).

## Polypharmacology

Given that fibrosis may develop from multiple stimuli, one approach is to explore polypharmacology, which has the capacity to simultaneously target multiple receptors and signaling cascades (79). Polypharmacology has the potential to produce higher efficacy drugs and reduced drug resistance (80). For example, one of the FDA-approved drugs for idiopathic lung fibrosis, nintedanib, simultaneously targets fibroblast growth factor receptors, platelet-derived growth factor receptors, and vascular endothelial growth factor receptors (81). With the advancements in GPCR structural biology and *in-silico* modeling, compounds can be rationally predicted, modified, and designed to target multiple receptors (41, 82).

## Identify more specific GPCR to minimize off-target effects

Another potential challenge for developing GPCR regulators for antifibrotic therapy is the widespread expression of these receptors among different cell types. Targeting the receptor may exert off-target effects. For example, PAR1 inhibition may not only attenuate myofibroblast activation, but also impair platelet function due to PAR1's high expression on platelets (49). Similarly, AR inhibition by caffeine inhibits liver fibrosis, but may simultaneously alter neurological states because of AR's high expression in neurons (83). Recent published single-cell RNA sequencing (sgRNA-seq) and single-nucleus RNA sequencing (snRNA-seq) datasets from human hearts demonstrated distinct fibrotic responses in various types of cardiomyopathies (28, 84–86). Therefore, multiple signaling pathways and crosstalk can be exploited, as they offer a rich blueprint of receptor distribution across different cell types and organs. By leveraging the single-cell sequencing database, we can identify GPCRs more specifically expressed on fibroblasts at a given tissue and upregulated during fibrosis to reduce potential off-target effects (28, 87).

## Conclusion

GPCR targeting approaches have yet to produce FDA-approved drugs for fibrotic diseases. Nevertheless, targeting GPCRs offers a tantalizing and promising therapy for treating cardiac fibrosis, as more GPCR-targeted antifibrotic therapies continue to enter clinical trials. Several challenges remain, and major questions need to be addressed before GPCR-mediated therapies for cardiac fibrosis are realized. The redundancy of profibrotic G protein signals and widespread GPCR expressions must be comprehensively considered to determine and refine our approach for antifibrotic therapy. A few solutions have been proposed to circumvent the challenges. Candidate drugs with polypharmacology or targeting common downstream signaling pathways have the potential to overcome the redundancy challenge.

On the other hand, more specific targets should be identified to minimize off-target effects by single-cell seq datasets of cardiomyopathy. The unique cardiac environment provides opportunities to target GPCRs highly specific expressed in the heart *via* crosstalk between fibroblasts, cardiomyocytes, endothelial cells, and ECM. Furthermore, the human induced pluripotent stem cell platform can be utilized as a high throughput and more complex human cellular model platform to complement animal models for drug development (88, 89). Collectively, these advances in our knowledge of GPCRs in cardiac fibrosis provide a unique perspective on the challenges and opportunities in exploring GPCRs to treat cardiac fibrosis.

## Author contributions

HZ and LR prepared the illustration, organized, and wrote the manuscript. RS wrote and edited the manuscript. All authors contributed to the article and approved the submitted version.

## Funding

This work was supported by American Heart Association 828308 (HZ).

## Conflict of interest

The authors declare that the research was conducted in the absence of any commercial or financial relationships that could be construed as a potential conflict of interest.

## Publisher's note

All claims expressed in this article are solely those of the authors and do not necessarily represent those of their affiliated organizations, or those of the publisher, the editors and the reviewers. Any product that may be evaluated in this article, or claim that may be made by its manufacturer, is not guaranteed or endorsed by the publisher.
